# Supplementary figures and images for: Expanded transcriptomic view of strawberry fruit ripening through meta-analysis
Source: PLoS One. 2021 Jun 1;16(6):e0252685. doi: 10.1371/journal.pone.0252685 (PMC8168840; doi:10.1371/journal.pone.0252685)

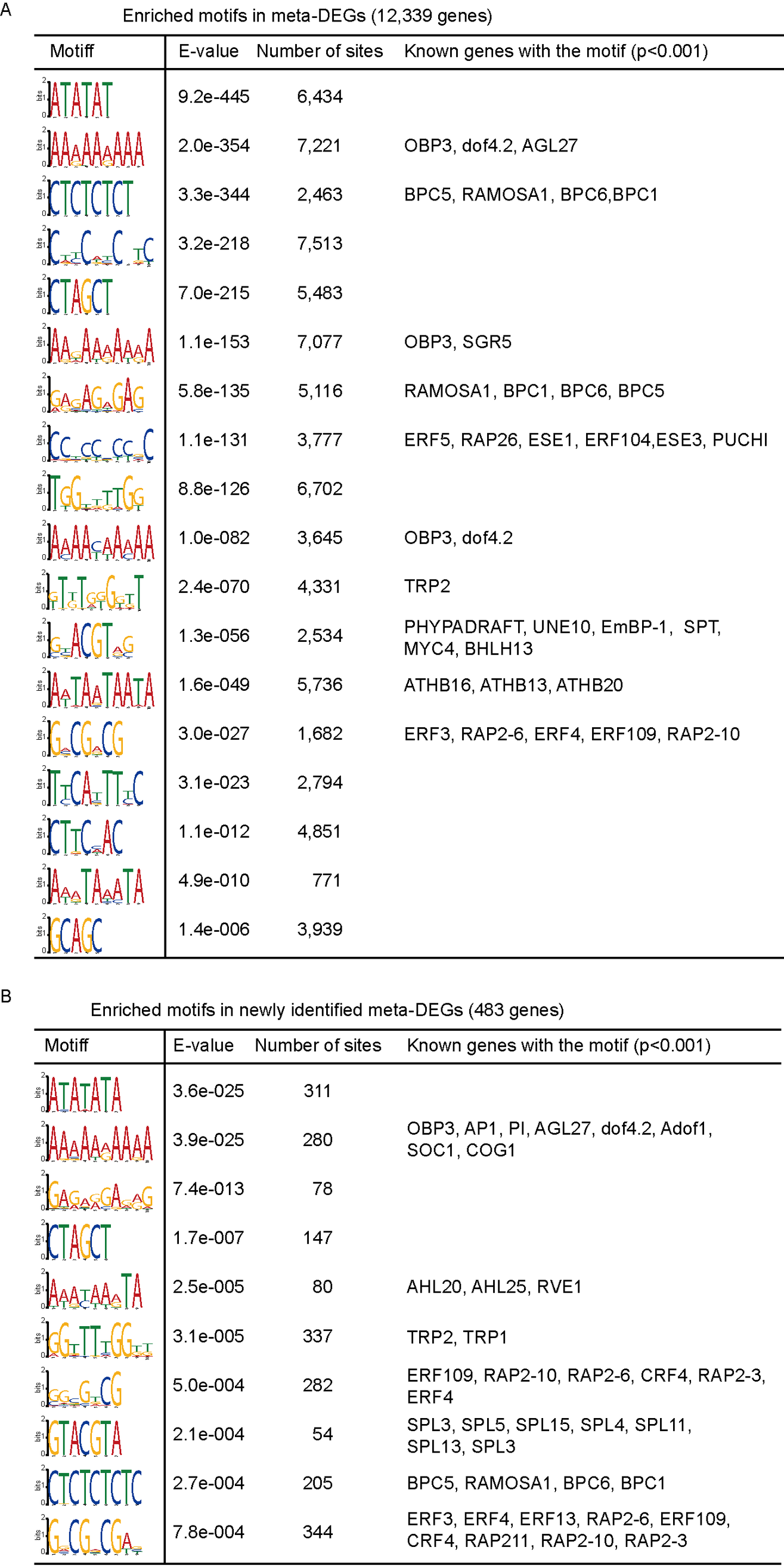

Supplement: S1 Fig — Enriched motifs sequences were searched in all meta-DEGs (12,339 genes) (A) and newly identified meta-DEGs (483 genes) (B). (TIF) [file pone.0252685.s002.tif]
