# Supplementary material for: Expanded transcriptomic view of strawberry fruit ripening through meta-analysis
Source: PLoS One. 2021 Jun 1;16(6):e0252685. doi: 10.1371/journal.pone.0252685 (PMC8168840; doi:10.1371/journal.pone.0252685)
Supplement: S1 Table — (DOCX) [file pone.0252685.s003.docx]

**S1 Table.** **RNA-Seq reads and mapping summary for studies used in this analysis.**

| Accession | Cultivar | Replicates | Raw reads | Filtered reads (a) | % | Mapped reads (b) | %  (b/a) | Mapped to gene (c) | %  (c/b) |
| --- | --- | --- | --- | --- | --- | --- | --- | --- | --- |
| PRJNA 394190 | Toyonoka | Large green 1 | 58,541,836 | 53,328,240 | 91.1 | 36,584,056 | 68.6 | 33,875,626 | 92.6 |
|  |  | Large green 2 | 60,581,866 | 54,986,252 | 90.8 | 37,760,622 | 68.7 | 32,769,190 | 86.8 |
|  |  | Red 1 | 61,847,198 | 56,937,822 | 92.1 | 39,069,454 | 68.6 | 32,959,024 | 84.4 |
|  |  | Red 2 | 59,579,024 | 54,770,646 | 91.9 | 37,320,084 | 68.1 | 34,407,266 | 92.2 |
| PRJNA 552213 | Benihoppe | Middle green 1 | 55,347,724 | 50,437,386 | 91.1 | 34,178,904 | 67.8 | 31,356,636 | 91.7 |
|  |  | Middle green 2 | 67,875,558 | 60,892,154 | 89.7 | 41,236,626 | 67.7 | 38,326,940 | 92.9 |
|  |  | Full red 1 | 65,177,098 | 58,815,334 | 90.2 | 37,470,140 | 63.7 | 34,088,014 | 91.0 |
|  |  | Full red 2 | 52,780,520 | 48,055,208 | 91.1 | 31,069,610 | 64.7 | 28,232,978 | 90.9 |
|  | Xiaobai | Middle green 1 | 50,123,738 | 44,176,420 | 88.1 | 29,186,780 | 66.1 | 27,244,660 | 93.3 |
|  |  | Middle green 2 | 59,381,530 | 53,532,906 | 90.2 | 35,731,206 | 66.7 | 33,001,564 | 92.4 |
|  |  | Full red 1 | 52,974,462 | 47,670,038 | 90.0 | 30,575,058 | 64.1 | 27,903,184 | 91.3 |
|  |  | Full red 2 | 56,987,370 | 51,517,796 | 90.4 | 33,707,606 | 65.4 | 30,425,488 | 90.3 |
|  | Snow princess | Middle green 1 | 50,821,198 | 46,161,258 | 90.8 | 31,436,540 | 68.1 | 28,879,912 | 91.9 |
|  |  | Middle green 2 | 62,943,910 | 56,714,686 | 90.1 | 38,695,118 | 68.2 | 35,628,920 | 92.1 |
|  |  | Full red 1 | 56,497,422 | 51,069,598 | 90.4 | 33,161,376 | 64.9 | 30,036,264 | 90.6 |
|  |  | Full red 1 | 50,084,180 | 45,429,274 | 90.7 | 29,065,230 | 64.0 | 26,307,982 | 90.5 |
| PRJNA 564159 | Kingsberry | Big green 1 | 51,481,300 | 48,633,002 | 94.5 | 32,829,042 | 67.5 | 28,926,534 | 88.1 |
|  |  | Big green 2 | 48,311,044 | 46,015,408 | 95.3 | 30,902,828 | 67.2 | 27,075,982 | 87.6 |
|  |  | Big green 3 | 48,358,878 | 46,927,134 | 97.0 | 30,880,476 | 65.8 | 26,276,564 | 85.1 |
|  |  | Fully red 1 | 54,893,984 | 52,397,894 | 95.5 | 35,110,716 | 67.0 | 30,422,956 | 86.6 |
|  |  | Fully red 2 | 61,083,170 | 58,682,182 | 96.1 | 38,807,690 | 66.1 | 33,182,262 | 85.5 |
|  |  | Fully red 3 | 46,005,284 | 43,809,596 | 95.2 | 30,024,984 | 68.5 | 26,293,208 | 87.6 |
|  | Sunnyberry | Big green 1 | 41,945,118 | 40,095,682 | 95.6 | 25,025,728 | 62.4 | 22,139,216 | 88.5 |
|  |  | Big green 2 | 41,153,956 | 39,362,892 | 95.7 | 24,767,000 | 62.9 | 22,109,282 | 89.3 |
|  |  | Big green 3 | 38,203,638 | 36,442,838 | 95.4 | 24,919,336 | 68.4 | 22,094,750 | 88.7 |
|  |  | Fully red 1 | 68,686,508 | 65,725,444 | 95.7 | 44,144,674 | 67.2 | 38,401,168 | 87.0 |
|  |  | Fully red 2 | 90,594,468 | 86,726,482 | 95.7 | 59,566,384 | 68.7 | 51,964,420 | 87.2 |
|  |  | Fully red 3 | 51,301,532 | 49,239,714 | 96.0 | 32,934,652 | 66.9 | 28,606,070 | 86.9 |
