# Supplementary material for: Expanded transcriptomic view of strawberry fruit ripening through meta-analysis
Source: PLoS One. 2021 Jun 1;16(6):e0252685. doi: 10.1371/journal.pone.0252685 (PMC8168840; doi:10.1371/journal.pone.0252685)
Supplement: S2 Table — (DOCX) [file pone.0252685.s004.docx]

**S2 Table. Pearson’s correlation coefficient (*R*^2^) between samples based on their expression profiles.**

|  | Toyo BG | Toyo FR | Beni BG | Beni FR | Xiao BG | Xiao FR | SnowBG | SnowFR | KingsBG | KingsFR | SunnyBG | SunnyFR |
| --- | --- | --- | --- | --- | --- | --- | --- | --- | --- | --- | --- | --- |
| Toyo BG |  | 0.02 | 0.30 | 0.14 | 0.58 | 0.13 | 0.46 | 0.20 | 0.40 | 0.13 | 0.21 | 0.15 |
| Toyo FR |  |  | 0.02 | 0.32 | 0.13 | 0.36 | 0.04 | 0.32 | 0.22 | 0.37 | 0.10 | 0.36 |
| Beni BG |  |  |  | 0.03 | 0.77 | 0.02 | 0.79 | 0.02 | 0.22 | 0.03 | 0.16 | 0.04 |
| Beni FR |  |  |  |  | 0.25 | 0.98 | 0.17 | 0.87 | 0.53 | 0.66 | 0.71 | 0.53 |
| Xiao BG |  |  |  |  |  | 0.23 | 0.80 | 0.24 | 0.51 | 0.20 | 0.39 | 0.20 |
| Xiao FR |  |  |  |  |  |  | 0.15 | 0.88 | 0.51 | 0.68 | 0.70 | 0.55 |
| Snow BG |  |  |  |  |  |  |  | 0.18 | 0.40 | 0.13 | 0.41 | 0.14 |
| Snow FR |  |  |  |  |  |  |  |  | 0.53 | 0.66 | 0.74 | 0.55 |
| Kings BG |  |  |  |  |  |  |  |  |  | 0.03 | 0.67 | 0.63 |
| Kings FR |  |  |  |  |  |  |  |  |  |  | 0.55 | 0.86 |
| SunnyBG |  |  |  |  |  |  |  |  |  |  |  | 0.51 |
| SunnyFR |  |  |  |  |  |  |  |  |  |  |  |  |
