# Supplementary material for: Expanded transcriptomic view of strawberry fruit ripening through meta-analysis
Source: PLoS One. 2021 Jun 1;16(6):e0252685. doi: 10.1371/journal.pone.0252685 (PMC8168840; doi:10.1371/journal.pone.0252685)
Supplement: S3 Table — (DOCX) [file pone.0252685.s005.docx]

**S3 Table.** **Number of DEGs in each study and DEGs retained after meta-analysis.**

| Cultivar | No. of DEGs | | No. of retained genes | Proportion of retained genes (%) |
| --- | --- | --- | --- | --- |
| Toyonoka | Total | 16,821 | 9,392 | 55.8 |
|  | Up | 5,867 | 2,490 | 42.4 |
|  | Down | 10,954 | 7,035 | 64.2 |
| Benihoppe | Total | 13,197 | 9,160 | 69.4 |
|  | Up | 4,424 | 2,380 | 53.8 |
|  | Down | 8,773 | 6,753 | 77.0 |
| Xiaobai | Total | 13,005 | 8,341 | 64.1 |
|  | Up | 5,152 | 2,431 | 47.2 |
|  | Down | 7,853 | 5,888 | 75.0 |
| Snow princess | Total | 17,184 | 9,367 | 54.5 |
|  | Up | 6,839 | 2,445 | 35.8 |
|  | Down | 10,345 | 6,816 | 65.9 |
| Kingsberry | Total | 5,664 | 4,476 | 79.0 |
|  | Up | 1,667 | 1,079 | 64.7 |
|  | Down | 3,997 | 3,361 | 84.1 |
| Sunnyberry | Total | 10,033 | 7,035 | 70.1 |
|  | Up | 3,252 | 1,744 | 53.6 |
|  | Down | 6,781 | 5,238 | 77.2 |
